# Supplementary figures and images for: The phospho-docking protein 14-3-3 regulates microtubule-associated proteins in oocytes including the chromosomal passenger Borealin
Source: PLoS Genet. 2022 Jun 6;18(6):e1009995. doi: 10.1371/journal.pgen.1009995 (PMC9203013; doi:10.1371/journal.pgen.1009995)

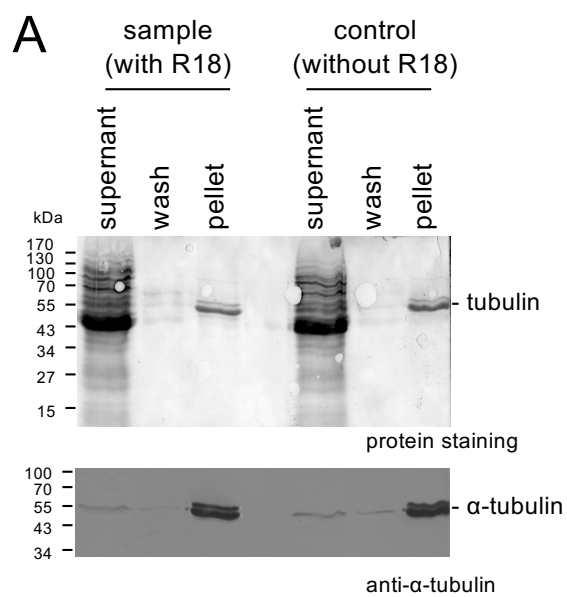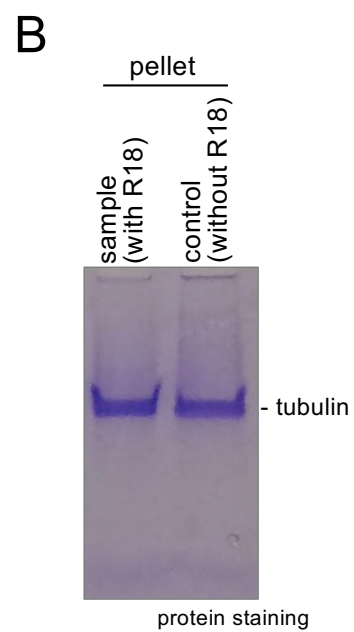

S1 Fig

Supplement: S1 Fig — (A) Microtubules and associated proteins were co-sedimented with or without the 14-3-3 inhibitor R18 from soluble extract of Drosophila ovaries. The original supernatant, wash of the original pellet, and the final pellet used for mass-spectrometry were analysed by western blot using an α-tubulin antibody and total protein staining. (B) The final pellets were run on SDS-PAGE and stained with Coomassie for mass-spectrometry. Nearly all tubulin in the extract was found in the pellet fraction, which predominantly consists of tubulin with minimal amounts of other proteins, regardless of the presence or absence of R18. (PDF) [file pgen.1009995.s001.pdf]

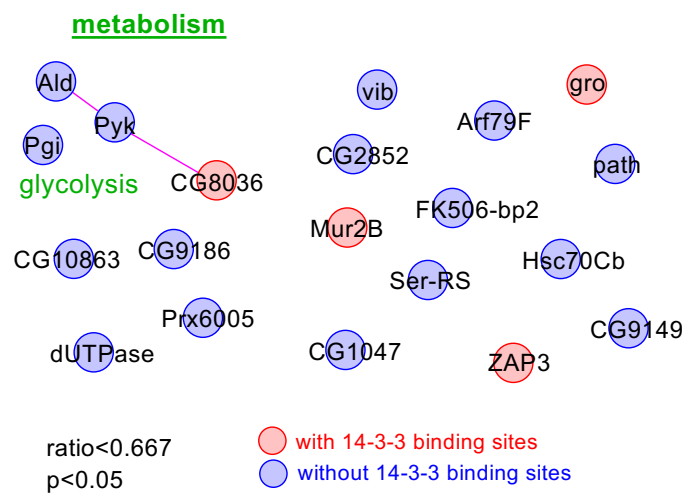

S2 Fig

Supplement: S2 Fig — Proteins with the fold change <0.6667 and the p<0.05 are shown. Red and blue indicate proteins with at least one predicted 14-3-3 binding site and without any, respectively. Lines indicate known physical interactions, and they do not have significantly more interactions than expected. (PDF) [file pgen.1009995.s002.pdf]

A

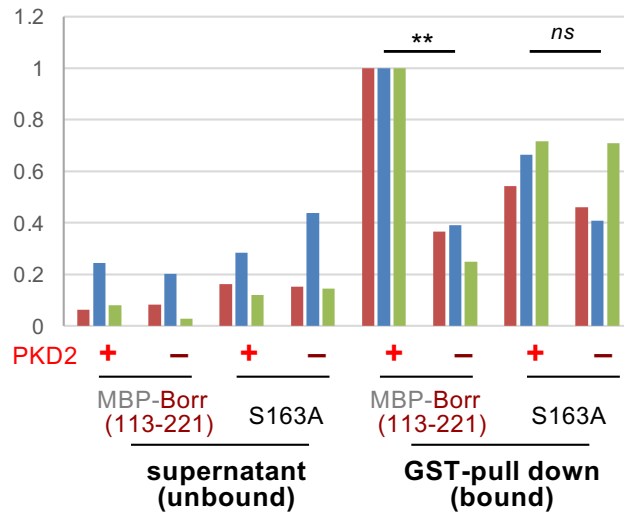

B

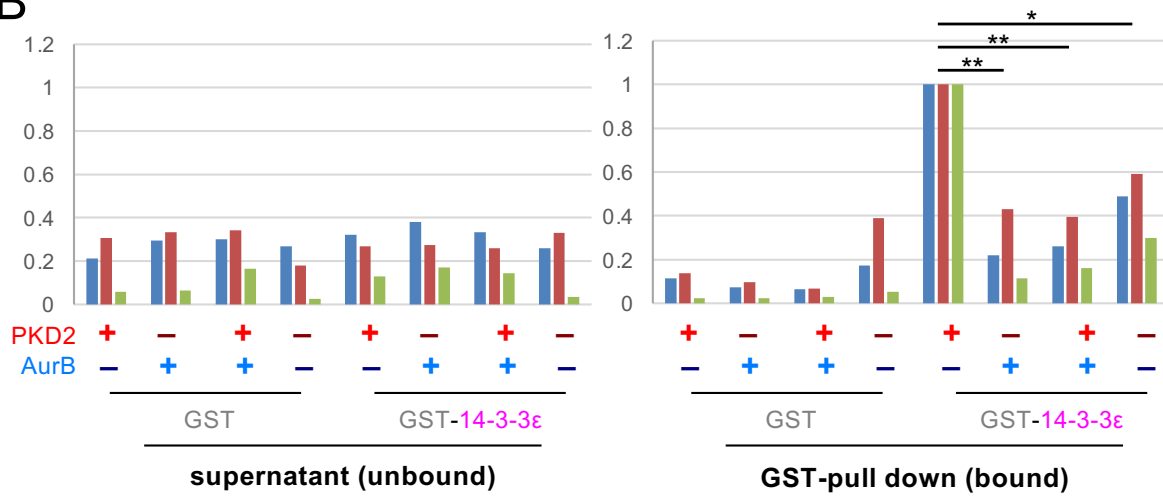

C

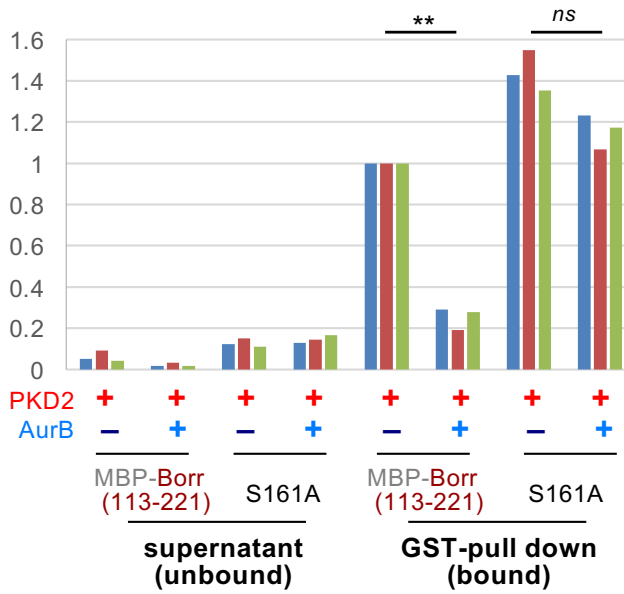

S4 Fig

Supplement: S4 Fig — Experiments shown in Fig 3D–3F were triplicated and presented as different coloured bars. The total signal intensities of the MBP-Borealin bands above the background were normalised to that of PKD2-phosphorylated MBP-Borr in GST-14-3-3 pull down in the same experiment. **, * and ns indicate p<0.01, p<0.05 and p>0.05, respectively. (A) MBP-Borealin(113–221) interacts with GST-14-3-3ε in a manner dependent on phosphorylation at S163. (B) An additional phosphorylation by Aurora B prevents PKD2-phosphorylated MBP-Borealin(113–221) from interacting with GST-14-3-3ε. (C) Aurora B cannot prevent interaction between GST-14-3-3ε and PKD2-phosphorylated MBP-Borealin(113–221) with S161A mutation. (PDF) [file pgen.1009995.s004.pdf]
