# Supplementary material for: The phospho-docking protein 14-3-3 regulates microtubule-associated proteins in oocytes including the chromosomal passenger Borealin
Source: PLoS Genet. 2022 Jun 6;18(6):e1009995. doi: 10.1371/journal.pgen.1009995 (PMC9203013; doi:10.1371/journal.pgen.1009995)
Supplement: S3 Fig — GST (A), GST-14-3-3ε (B) MBP-Borr(113–221) (C), MBP-Borr(113–221,S163A) (D) and MBP-Borr(113–221,S161A) (E) were affinity-purified using columns containing S-glutathione beads or amylose beads and eluted in buffer containing glutathione or maltose, respectively, as described in Materials and Methods. Purification intermediates were analysed by SDS-PAGE and stained with Coomassie. (PDF) [file pgen.1009995.s003.pdf]

A GST purification

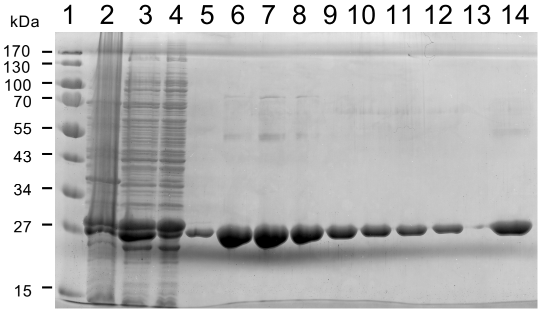

1. ladder 5 µL
2. total cell lysate 10 µL
3. cleared lysate 10 µL
4. unbound lysate 10 µL
5. fraction 1, 10 µL
6. f2 10 µL
7. f3 10 µL
8. f4 10 µL
9. f5 10 µL
10. f6 10 µL
11. f7 10 µL
12. f8 10 µL
13. buffer only
14. concentrated fractions 2-4

B GST-14-3-3ε purification

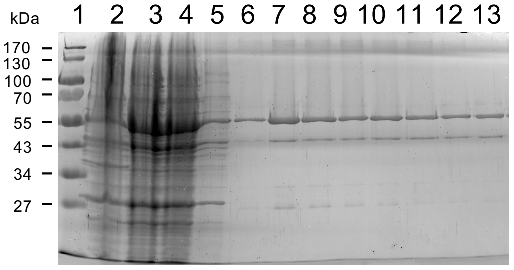

1. ladder 5 µL
2. total cell lysate 10 µL
3. cleared lysate 10 µL
4. unbound lysate 10 µL
5. fraction 1, 10 µL
6. f2 10 µL
7. f3 10 µL
8. f4 10 µL
9. f5 10 µL
10. f6 10 µL
11. f7 10 µL
12. f8 10 µL
13. concentrated fractions 3-7 10 µL

C MBP-Borr(113-221) purification

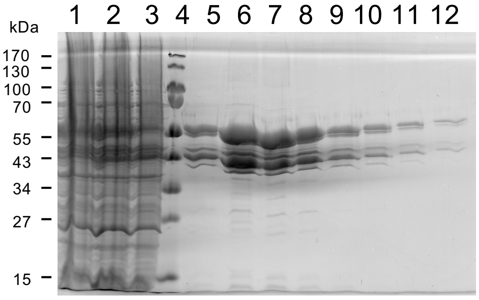

1. total cell lysate 10 µL
2. cleared lysate 10 µL
3. unbound lysate 10 µL
4. ladder 5 µL
5. fraction 1, 10 µL
6. f2 10 µL
7. f3 10 µL
8. f4 10 µL
9. f5 10 µL
10. f6 10 µL
11. f7 10 µL
12. f8 10 µL

D MBP-Borr(113-221,S163A) purification

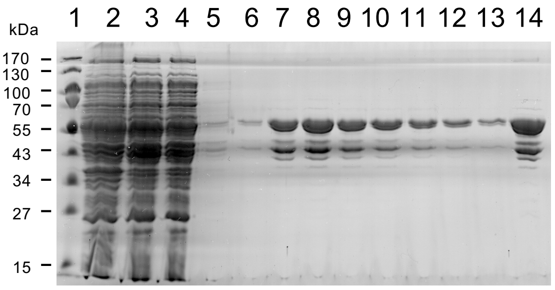

1. ladder 5 µL
2. total cell lysate 10 µL
3. cleared lysate 10 µL
4. unbound lysate 10 µL
5. wash
6. fraction 1, 10 µL
7. f2 10 µL
8. f3 10 µL
9. f4 10 µL
10. f5 10 µL
11. f6 10 µL
12. f7 10 µL
13. f8 10 µL
14. Concentrated fractions 2-4 10 µL

E MBP-Borr(113-221,S161A) purification

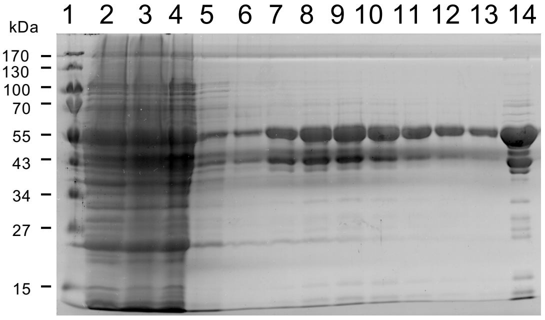

1. ladder 5 µL
2. total cell lysate 10 µL
3. cleared lysate 10 µL
4. unbound lysate 10 µL
5. wash
6. fraction 1, 10 µL
7. f2 10 µL
8. f3 10 µL
9. f4 10 µL
10. f5 10 µL
11. f6 10 µL
12. f7 10 µL
13. f8 10 µL
14. Concentrated fractions 2-4 10 µL

S3 Fig
