# Supplementary material for: The phospho-docking protein 14-3-3 regulates microtubule-associated proteins in oocytes including the chromosomal passenger Borealin
Source: PLoS Genet. 2022 Jun 6;18(6):e1009995. doi: 10.1371/journal.pgen.1009995 (PMC9203013; doi:10.1371/journal.pgen.1009995)
Supplement: Attachment — Submitted filename: revision letter Repton.pdf [file pgen.1009995.s017.pdf]

***PLoS Genetics manuscript PGENETICS-D-21-01648***

Dear Kim and Greg

We are pleased to have received enthusiastic and constructive comments from all the reviewers. Following your advice, we have incorporated the reviewers' comments by mostly improving the presentation of results in the manuscript. In addition, due to the time constraint, we have focused our efforts on carrying out the most crucial experiment showing that S163A mutation indeed disrupts interaction of Borealin with 14-3-3 *in vitro*. S161A mutation has not been tested, but as we have shown that S163 is the 14-3-3 binding site and there are no other serine or threonine near it (plus/minus 9 amino acids), the Aurora B effect on 14-3-3 binding is unlikely be due to phosphorylating other sites than S161. Incorporating all reviewers' comments has greatly improved the manuscript, which we believe is now suitable for publication in *PLoS Genetics*.

With kind regards

Hiro

Hiroyuki Ohkura

Hiro Ohkura

Professor of Cell Biology and Wellcome Investigator in Science
Wellcome Centre for Cell Biology
The University of Edinburgh
Edinburgh EH9 3BF, UK
+44-131-650-7094
h.ohkura@ed.ac.uk

**[Summary of the revision]**

**New experimental data and analysis added**

- 14-3-3 pull down *in vitro* using Borealin S163A mutant
- quantification of band intensities in Fig 3
- the names and interactions of proteins that showed significantly lower amounts in the microtubule fraction under 14-3-3 inhibition as a supplementary figure (S2 Fig)
- purification of MBP-Borr(113-221, S163A) in S3 Fig

**Revisions of the text and presentation.**

- adding more background information on *Drosophila* 14-3-3 in Introduction and Results
- mentioning that the kinase for S163 is unknown

• stating that S161 and S163 are phosphorylated in vivo
• appending “epsilon” to “14-3-3” in experimental description
• stating reduction of the GFP signal at centromeres and the spindle in Borr phospho-mutants
• clarifying the relative intensities of the Rcc1-mCherry signal
• clarifying the relative amounts of GFP-Borr
• discussing an alternative hypothesis for Borealin regulation
• discussing potential combinatorial effects due to possible 14-3-3 binding sites in Incenp
• correcting over-interpretation of the role of regulating microtubule binding
• adding discussion on possible implication for cytokinesis
• correcting typos/small words
• correcting the legend of Figure 1
• quantifying the band intensities in Figure 3
• correcting the legend of Figure 4
• reordering the data in S1 Table by ratio
• adding two separate sheets to S1 Table, only containing proteins that significantly increased or decreased their microtubule binding under 14-3-3 inhibition
• defining mass spec data terminology in the legends of supplementary tables

**Reviewer #1:**

*This study addresses an important problem and reports interesting findings that advance the field.*

**We are very pleased to hear very positive comments and constructive suggestions which we have incorporated to the revised manuscript as follows.**

**Specific points**

***1.** I suggest that they use the Borealin S163A (non-phoshorylatable) mutant in the pull-down experiments*

**Although only one predicted 14-3-3 binding site in this short fragment (113-221), we agree that this is a crucial experiment to test whether 14-3-3 binds to phosphorylated S163. We carried out 14-3-3 *in vitro* pull down experiments using an S163A mutant. As expected, the mutation greatly decreased the interaction with 14-3-3 in the presence of PKD2. This has been confirmed by triplicated experiments. The data has been added to Figure 3.**

***2.** The mass spectrometry data is likely to be a useful resource for the field and is a strength of the paper. Because of this, I think it would be helpful for the authors to better organize and present these data, so that readers can more easily analyze it.*

**We agree. We have added legends to briefly explain the terminology of mass spec data, and reordered S1 Table according to the ratio. Excel files allow other researchers to reorder, depending on the purpose of their analysis.**

***3.** Table S1 in particular reports data that the field will be interested in. However, I think that it could be better organized...*

**We reordered the data in the S1 Table according to the ratio. Also we have added two separate sheets to S1 Table, each of which contains only proteins that significantly increased or decreased their microtubule binding under 14-3-3 inhibition.**

***4.** The legend for Figure 1 has an error (it says there is a protein gel in Figure 1B, but this is not the case).*

**Thank you very much for pointing out this error. We removed it from the legend.**

***5**. it would be useful for the authors to expand upon and clarify their model*

**Yes, indeed the phospho-mutations appear to affect Borealin localisation to both centromeres and spindle microtubules. We have two main hypotheses. One is (as the reviewer suggested) that 14-3-3 regulates localisation to both centromeres and spindle microtubules. The other is that 14-3-3 inhibits Borealin binding to cytoplasmic microtubules, which could sequester Borealin and reduce the effective concentration of Borealin near chromosomes. In the original manuscript, we only discussed the second hypothesis. We have expanded discussion, including the first hypothesis too.**

***6.** It would be helpful for the reader if there was more background information on Drosophila 14-3-3 proteins. ... What is the other isoform called? Have people tried to deplete it, and if so, was there a phenotype? In the authors' previous study on NCD, did they assess both isoforms? Adding this type of broader background information would help people not familiar with the 14-3-3 literature better understand the current study. Additionally, in the results section the authors mention an inhibitor that they predict inhibits both isoforms. Having more information about the isoforms (e.g. how similar they are) and about the inhibitor would help the reader evaluate this claim.*

**Our previous study showed that knockdown of one of the two 14-3-3 isoforms (ε) alone caused the spindle defect in oocytes. Although knockdown of the other isoform (ζ) alone showed no defects, double knockdown with 14-3-3ε resulted in much more severe spindle defects than 14-3-3ε knockdown alone. The 14-3-3 inhibitor R18 has been shown to inhibit various isoforms including ε and ζ in vertebrates. The sequence identity (>80%) between the same isoforms in *Drosophila* and vertebrates is higher than the identity (60-70%) between**

**different isoforms. Therefore we expect that the inhibitor inhibits both isoforms (ε and ζ) in *Drosophila*. This information is now included in Introduction and Results.**

***7.*** *I think it would be helpful to quantify the intensity of the bands on the gels in Figure 3 to give a more quantitative view of how much protein is pulled down.*
**We have quantified them as requested. It is a tricky pull-down involving quick washes, as generally 14-3-3 interacts with substrates with a low affinity (Kd is in µM range). In addition, the absolute signals on each western blot vary. Therefore, we always process the samples including a control in parallel and run on the same gel, and compare only between them. Triplicated experiments all showed the same clear differences, although the absolute intensities are different between the experiments.**

***8.*** *This reviewer was left wondering which kinase phosphorylates S163.*
**We do not think the *Drosophila* orthologue of PKD2 is a good candidate, as RNAseq showed that it has no or extremely low expression in ovaries. We do not know the identity of the kinase for S163 yet. This information has been added.**

*Typos/small wording changes:*
**Thank you very much for pointing these out. We have corrected them.**

**<u>Reviewer #2</u>**
*...the authors' development of a new assay for the identification of MT-binding proteins in ovary extracts, which I found impressive.... I will be very supportive of publication after consideration of the following points:*
**We are delighted and grateful for the strong support of publication.**

***1***\- *Do failures in the mechanisms proposed affect egg viability?*
**We expect that it affects egg viability (fertility). We have tried to do egg viability assay, but as the reviewer pointed out, also in our hands this driver (V37) gave a high rate of embryonic lethality even without RNAi.**

*2- It would be interesting to test if the same mechanism also affects the ability of Borr to localize to centromeres.*
**Both centromere and spindle signals looked reduced, although it is difficult to quantify the signal intensities separately. The text in Results has been clarified accordingly.**

*3- The Western shown in Fig 4C is not clear enough.*
**This is a tricky western of ovary samples, as the GFP-Borr signal is not strong. Here we are not claiming exactly the same amount. Instead, differences in the signal intensity are unlikely to be explained simply by the relatively small differences in the amounts of GFP-Borr in ovaries. We have modified the text accordingly.**

*4- It would be good to test it with the GFP-Borr transgenic flies... To this end, could the authors measure the fluorescence intensity of their different variants of GFP-Borr on MTs of the polar body?*
**The signals of cytoplasmic microtubules were too weak to be visible reliably. Looking at polar bodies (that are associated with microtubule bundles) is potentially a good idea, but as the oocytes we use are arrested in metaphase I, polar bodies are not formed yet.**

*5- In all biochemical experiments, 14-3-3e (epsilon) is used and never 14-3-3z (zeta). Do the authors assume that 14-3-3z would behave in the similar way? This should at least be discussed if not tested. Also, the name 14-3-3e should be used throughout when referring to the protein used in experiments.*

**We do not know whether this interaction is isoform-specific. To aid clarity, an isoform (14-3-3ε) is now specified throughout to describe the results.**

*6- Results in Fig 3E suggest that 14-3-3e may bind Borr to some degree when unphosphorylated. Would the authors like to comment on it? It looks like this basal binding may be also inhibited by phosphorylation by AurB.*

**As the amount pulled down also increased slightly with GST alone, this suggests it may have some affinity to GST, though we do not know why. We do not think there is biological significance.**

*7- Do the authors think that PDK2 may be a kinase that contributes to phosphorylate S163 in vivo? Can they speculate on what other kinases may be involved?*

**We do not think the *Drosophila* orthologue of PKD2 is a good candidate, as RNAseq showed that it has no or extremely low expression in ovaries. We do not know the identity of the kinase for S163 yet. The information has been added.**

*8- I wonder if the regulation of Borr/CPC by 14-3-3 plays a role in cytokinesis. We could imagine that 14-3-3 binding helps prevent CPC binding to the MTs until cytokinesis, when it is recruited to the central spindle. A phosphatase could dephosphorylate S163 during mitotic exit to promote this transition. This may be an idea for future experiments and/or a discussion point.*

**We agree that the same mechanism may operate during cytokinesis when the CPC accumulated to the midbody. As our proposed mechanism can provide a self amplification loop, in theory this can confer a localised switch-like property to the CPC activity during cytokinesis and/or enhance the accumulation of the CPC to a tighter region. We have included this in Discussion.**

*9- Fig 1B mentioned in the legend (Protein gel) is missing.*

**Thank you very much for pointing this out. We have now corrected it.**

**Reviewer #3:**

*I appreciate the differential screening strategy... Unfortunately, the link between the initial screen for MAPs and the final result on the centromeric de-localization of Borealin are unclear. Finally, several results are correlative and essential controls are missing. The mechanism is speculative.*

**We have carried out additional experiments (test S163A for 14-3-3 pulldown) to fill a gap and revised the text etc by incorporating the suggestions as described below.**

*Main concerns.*

***1**-It is essential to show that S161 and S163 of Borealin are essential for interaction with 14-3-3.*

**Although only one predicted 14-3-3 binding site in this short fragment (113-221), we agree that this is a crucial experiment to test whether 14-3-3 binds to phosphorylated S163. We carried out 14-3-3 *in vitro* pull down experiments using an S163A mutant. As expected, S163A mutation greatly decreased the interaction with 14-3-3 in the presence of PKD2. This has been confirmed by triplicated experiments. The data has been added to Results and Figure 3.**

**We could not make or test S161A mutation due to the time constraint. However, as we have shown that S163 is the 14-3-3 binding site and there are no other serine or threonine near it (plus/minus 9 amino acids), the Aurora B effect on 14-3-3 binding is unlikely be due to phosphorylation of sites other than S161.**

*Other important concerns that nevertheless need to be amended.*
***1**-This is a matter of taste but I believe too much emphasis is made on the previous Ncd publication. The mechanism involved may be completely different for Borealin.*
**We think it is likely that they share the common mechanism involving 14-3-3 and Aurora B, but do not exclude the possibility that they regulate Borealin differently from Ncd. The Discussion has been revised to include this possibility.**

***2**- Would it be possible to also include in figure 2 the identity of the proteins that are less present in the MT pellet in the presence of the R18 inhibitor.*
**Their names, protein-protein interactions and presence/absence of predicted 14-3-3 binding sites of these proteins have been included as a supplementary figure (S2 Fig). As you can see, they are not enriched in known interactions, spindle/microtubule functions or 14-3-3 binding sites.**

***3)** L611. Where is the gel in the figure 2.*
**Thank you pointing out the error in the figure legend. We have corrected it.**

***4)** RCC1 levels are not comparable and significantly different (there is a discrepancy between Lane 283 and Figure 4B lower panel). Is it because of the image acquisition? Is it because the S161 mutant triggers loss of RCC1 targeting?*
**Due to the thickness of oocyte samples, the measurement of signal intensity can be substantially affected by the depth of the spindle location within oocytes. We monitor these potential depth effects by simultaneously measuring Rcc1-mCherry and GFP-Borr signal intensity. In this case, the measured intensities of Rcc1-mCherry in Borealin phospho-mutants were not significantly different from that in wild-type Borealin. We have clarified the text accordingly. The Rcc1-mCherry signal in S163A was significantly stronger than that in S161A. However, as the measured GFP-Borr(S163A) signal was significantly weaker than GFP-Borr(S161A), it does not affect our conclusion.**

***5)** The images of IF acquisitions are very small and it is difficult to see the differences. Scale bar are missing in some panels.*
**We have enlarged the images. Thank you for pointing out a missing scale bar; we have added it accordingly.**

***6)** Western Blots are not of optimal quality. Load same amounts of proteins (Figure 4C)*
**This is a tricky western of ovary samples, as the GFP-Borr signal is not strong. Although there are some smears, similar amounts of proteins appeared to be loaded in each lane judged from yolk proteins (the strong bands at 43kDa) and minor protein bands below them. Here we are not claiming exactly the same amount. Instead, the differences in the signals are unlikely to be explained by the observed differences in the amounts of GFP-Borealin in ovaries. We revised the text accordingly.**

***7)** Figure 3. Please provide quantitation, I do not see differences in Fig 3D between ++ and +. Alternatively, use one condition.*
**As requested, the band intensities have been quantified and included in Figure 3. Using different amounts of kinase was intended to confirm that Borealin is fully phosphorylated, not to show different levels of phosphorylation. In any case, this figure has been replaced by a result from a new experiment using a phospho-mutant S163A and a single concentration of PKD2.**

***8)** Figure 3 E. There is more MBP-Bor bound to GST-14-3-3 in the presence of PkD2 (well ° 5) but there is obviously more GST14-3-3 on the beads. See previous comment (well 8) There seems to be more bound*

*MBP-Bor to GST-13-3-3 without any of the 2 Kinases, how it is possible?*

**As the amount pulled down also increased with GST alone, suggesting it may have some affinity to GST (not to 14-3-3) for some reason, we do not think that it has a biological significance.**

***9)*** *What is the evidence that regulation of interactions between 14-3-3 and Borealin (by PKD2 and Aur B) is regulated via Ser161 and S163. Are these sites phosphorylated in vivo? (See my main concern N°1).*

**Indeed, both S161 and S163 are phosphorylated *in vivo*, according to previous phosphoproteomics studies in *Drosophila* (Hu et al, 2019). This information has been included in Results. We have also carried out an experiment, showing that non-phosphorylatable S163A mutation greatly decreased 14-3-3 interaction *in vitro*. This has been confirmed by triplicated experiments. The data has been added to Results and Figure 3.**

***10****) There is also a 14-3-3 binding site in INCENP. Please comment for potential combinational effects on CPC localization.*

**We have pointed out potential combinational effects in Discussion.**

***11****) L667. Arrowheads are not visible on the figure.*

**Thank you for pointing this out. The legend has been revised accordingly.**

***12)*** *In a previous study this team has shown that the CPC regulates MT nucleation on the meiotic spindle. However, MTs appear to be well nucleated in the absence of Borealin that is a member of the CPC complex. This is should be clarified.*

**There are two possible explanations for this: either RNAi results in only partial depletion of Borealin or Borealin is not essential for microtubule nucleation in oocytes. To clarify this, we have made two more transgenes to express different shRNA against Borealin in oocytes, but spindle bipolarity was not affected by the expression of either shRNA. As we could not exclude either possibility, both possibilities are pointed out in Results.**

***13****) Usually, the RNAi/Rescue system is validated by Western Blot to challenge depletion of the endogenous protein and persistence of the rescuing tagged protein. This is not shown in this study. Please provide evidence the system is working. Is there a Borealin antibody to validate the strategy?*

**We agree that we do not know how much Borealin was depleted by RNAi and how much transgene was expressed relative to the endogenous protein, as we do not have a Borealin antibody. However, as geneticists, we consider that rescue of RNAi phenotype by an RNAi-resistant wild-type transgene is a good (more direct) evidence the system is working.**

***14****) There is confusion between spindle and centromere localization. The screen aimed to find proteins for which 14-3-3 binding abolishes interaction with meiotic MTs. However the Borealin protein (and possibly other CPC subunits) is located on the centromeres and not on the meiotic spindle.*

**In *Drosophila* oocytes, the McKim lab and our lab showed that CPC subunits (at least Aurora B, Incenp and Borealin) are located on the meiotic spindle, as well as the centromeres in meiotic metaphase I. This spindle localisation in meiotic metaphase I is distinct from that in mitotic cells, and likely plays an important role in assembly and stability of the bipolar spindle without centrosomes in oocytes.**

***15****) Lane 365-366-“ Our study identified a novel mechanism regulating microtubule binding activity of the CPC, which is essential for bi-orienting homologous chromosomes in oocytes. » There are no direct evidences that MT binding activity of Bor is involved in CPC centromere localization (S161 and S163 appears important). This is over interpretation.*

**We agree that this is over-interpretation. We have revised the text accordingly. Thank you for pointing this out.**
