# Supplementary material for: The phospho-docking protein 14-3-3 regulates microtubule-associated proteins in oocytes including the chromosomal passenger Borealin
Source: PLoS Genet. 2022 Jun 6;18(6):e1009995. doi: 10.1371/journal.pgen.1009995 (PMC9203013; doi:10.1371/journal.pgen.1009995)
Supplement: Attachment — Submitted filename: revision letter Rev2.pdf [file pgen.1009995.s018.pdf]

PLoS Genetics manuscript PGENETICS-D-21-01648

Dear Kim and Greg

We are pleased that two of the reviewers strongly support publication, but the third reviewer requested two experiments, the S161A pulldown and GFP-Borr western. We are thankful to the Editors for giving an opportunity to carry out these experiments. We are glad to tell that the experiments have been successful, and hope that inclusion of the results has made the manuscript suitable for publication in *PLoS Genetics*.

Hiro

Hiro Ohkura
Professor of Cell Biology and Wellcome Investigator in Science
Wellcome Centre for Cell Biology
The University of Edinburgh
Edinburgh EH9 3BF, UK
+44-131-650-7094
h.ohkura@ed.ac.uk

[Summary of the revision]

New experimental data and analysis added

- 14-3-3 pull down *in vitro* using Borealin S161A mutant (Fig 3F)
- quantification and statistical analysis of triplicated pull-down experiments (S4 Fig)
- western blot of MBP-Borr and its variants in ovaries (Fig 4C).
- purification of MBP-Borr(113-221, S161A) (S3 Fig E)

Revisions of the text and presentation.

- reworded to "This is likely to reflect partial depletion of Borealin, although we cannot exclude the possibility that Borealin is not essential for normal spindle morphology."
- reworded to "a self amplification loop by which the CPC promotes further recruitment of the CPC".

[Point-by-point response]

Reviewer #1:

The manuscript by Repton et.al. is substantially improved and the authors did a good job addressing my concerns. The manuscript is now more accessible to a broad audience, and I think will be of interest to the readers of PLOS Genetics.

Thank you very much for helping us to improve the manuscript.

I found one small typo but otherwise have no further suggestions
Line 204: "which increases or decreases" should be "that increase or decrease"

Thank you. Corrected.

Reviewer #2:

My points have been addressed in an acceptable manner. I am in favor of accepting the revised manuscript for publication.

Thank you very much for helping us to improve the manuscript.

Reviewer #3:

"I had requested a number of additional experiments. Despite the changes in the manuscript, I note that the authors did not perform any of these experiments."

"I still have serious concerns because none of the experiments I requested have been performed."

These statements are based on a false impression. For the first revision, we had spent nearly two months to carry out one of the requested experiments (S163A in 14-3-3 pull down).

It is all the more unfortunate that the most complicated experiment (in vivo study) was performed... This is detrimental to the quality of the manuscript and I think that the in vitro experiment with the S161A mutant would have really provided the missing piece of the puzzle to make a solid story.

***An in vitro* experiment involving the multiple protein components is not trivial. As it often takes much more time than anticipated, we were reluctant to request to set a new revision deadline. This time we were very fortunate that the S161A experiment worked in the first try. The triplicated experiments showed that Aurora B cannot prevent 14-3-3 interaction with Borealin(S161A). We agree that this result has made the story complete. This data has been added as Fig 3F.**

If the experiment in Fig 3 was performed 3 times, there should be some quantification with appropriate tests (n=3, P<, SD…etc).

We have now quantified each of the triplicated experiments and carried out statistical analysis, which confirms our conclusion. These data have been included as a supplementary figure (S4 Fig).

. "S2 Fig). Among the 47 proteins that increased microtubule binding in the presence of the 14-3-3 inhibitor, we found 48 known protein-protein interactions among these 47 proteins, which is significantly higher than expected from a random set of 47 proteins (17 interactions; p=1.2e-09) » How this is possible ?

We are not sure why and where the reviewer did not think this possible. Among 47 proteins, a maximum of 1,128 and a minimum of 0 protein-protein interactions are possible. 48 and 17 interactions are both well within this range. The large difference of these two numbers seems to justify this low p-value.

. Lane 308. The authors are entangled with their signal quantifications, (Western Blots and IF images). Please clarify. The authors claim The RCC1 levels are not different (this is not reflected by the statistical tests).

According to a statistical test (Wilcoxon rank sum test), levels of the Rcc1 signals in either mutant is not significantly different (p>0.05) from that in wild type, although they are significantly different between the two mutants.

I would suggest to use RCC1 as a probe to position the meiotic spindle and to stick on GFP signal quantification.

We are not sure what this suggestion means. If this means either using Rcc1 signal to normalise the data or to select a subset of the data, we prefer presenting raw data to readers rather than "adjusted" data.

However, this is possible only if the proteins are expressed are similar levels and if the GFP blot is OK. As I mentioned in my previous review the anti-GFP Western blot is far from optimal, I suggest using another GFP antibody, (or performing IP using GFP TRAP beads) and load similar amounts of proteins. It is not an unjustified request to ask for quality Western blots for a high-quality journal like PLOS Genetics.

It has been difficult to get the exact amount of loading and good quality of signals in all 4 samples. We have tried 8 times, including use of a lower-percentage gel which gave a better quality of the signals. We replaced Fig 4C by the best western blot we have obtained.

. Lane 409-411. ...I fail to understand the self-amplification loop mechanism, please explain.

We have reworded to "a self amplification loop by which the CPC promotes further recruitment of the CPC".
